# *Basidiobolus omanensis* sp. nov. Causing Angioinvasive Abdominal Basidiobolomycosis

**DOI:** 10.3390/jof7080653

**Published:** 2021-08-12

**Authors:** Abdullah M. S. Al-Hatmi, Abdullah Balkhair, Ibrahim Al-Busaidi, Marcelo Sandoval-Denis, Saif Al-Housni, Hashim Ba Taher, Asmaa Hamdan Al Shehhi, Sameer Raniga, Maha Al Shaibi, Turkiya Al Siyabi, Jacques F. Meis, G. Sybren de Hoog, Ahmed Al-Rawahi, Zakariya Al Muharrmi, Ahmed Al-Harrasi, Badriya Al Adawi

**Affiliations:** 1 Natural & Medical Sciences Research Center, University of Nizwa, Nizwa 616, Oman; saifalhousni@unizwa.edu.om (S.A.-H.); ahmed@unizwa.edu.om (A.A.-R.); aharrasi@unizwa.edu.om (A.A.-H.); 2 Department of Biological Sciences & Chemistry, College of Arts and Sciences, University of Nizwa, Nizwa 616, Oman; 3 Centre of Expertise in Mycology, Radboud University Medical Centre/Canisius Wilhelmina Hospital, 6532 SZ Nijmegen, The Netherlands; jacques.meis@gmail.com (J.F.M.); sybren.dehoog@radboudumc.nl (G.S.d.H.); 4 Infectious Diseases Unit, Department of Medicine, Sultan Qaboos University Hospital, Muscat 123, Oman; balkhair2020@gmail.com (A.B.); ibrahimbusaidi@gmail.com (I.A.-B.); Bataher84@gmail.com (H.B.T.); 5 Westerdijk Fungal Biodiversity Institute, 3584 CT Utrecht, The Netherlands; m.sandoval@wi.knaw.nl; 6 Department of Pathology, Sultan Qaboos University Hospital, Muscat 123, Oman; Asmaah@squ.edu.om; 7 Department of Radiology and Molecular Imaging, Sultan Qaboos University Hospital, Muscat 123, Oman; sameerraniga@yahoo.com; 8 Department of Surgery, Sultan Qaboos University Hospital, Muscat 123, Oman; maha.alshaibi@squ.edu.om; 9 Department of Microbiology and Immunology, Sultan Qaboos University Hospital, Muscat 123, Oman; dr.alsiyabi@gmail.com (T.A.S.); almuharrmi@gmail.com (Z.A.M.); 10 Departments of Medical Microbiology and Infectious Diseases, Canisius Wilhelmina Hospital, 6532 SZ Nijmegen, The Netherlands

**Keywords:** gastrointestinal, basidiobolomycosis, phylogeny, ITS, LSU, morphology, *Basidiobolus*

## Abstract

Human infectious fungal diseases are increasing, despite improved hygienic conditions. We present a case of gastrointestinal basidiobolomycosis (GIB) in a 20-year-old male with a history of progressively worsening abdominal pain. The causative agent was identified as a novel *Basidiobolus* species. Validation of its novelty was established by analysis of the partial ribosomal operon of two isolates from different organs. Phylogeny of ITS and LSU rRNA showed that these isolates belonged to the genus *Basidiobolus,* positioned closely to *B. heterosporus* and *B. minor*. Morphological and physiological data supported the identity of the species, which was named *Basidiobolus omanensis*, with CBS 146281 as the holotype. The strains showed high minimum inhibitory concentrations (MICs) to fluconazole (>64 µg/mL), itraconazole and voriconazole (>16 µg/mL), anidulafungin and micafungin (>16 µg/mL), but had a low MIC to amphotericin B (1 µg/mL). The pathogenic role of *B. omanensis* in gastrointestinal disease is discussed. We highlight the crucial role of molecular identification of these rarely encountered opportunistic fungi.

## 1. Introduction

Basidiobolomycosis is a rare infection in humans that usually affects skin and subcutaneous tissues of limbs and trunk. The disorder prevalently occurs in immunocompetent individuals. The first case of subcutaneous basidiobolomycosis caused by *Basidiobolus ranarum* was reported from Indonesia in 1956 [1]. Prior to this report, van Overeem [2] described a case in a horse by an as yet unidentified *Basidiobolus* species. Infections have also been reported in dogs [3]. Human gastrointestinal basidiobolomycosis (GIB) is increasingly recognised in immunocompromised or otherwise debilitated patients [4,5,6,7]. This deep infection, recently described from the Middle East [7], may take a serious course. The authors analysed 28S rDNA sequences of five strains and found them to cluster in a monophyletic branch close to *B. haptosporus*, having 99.97% similarity with the latter species and slightly lower (99.25%) with *B. ranarum.* Given the low variability of the used marker, these results suggest a possibly undescribed *Basidiobolus* species.

*Basidiobolus* has classically been listed as a member of the phylum Zygomycota. On the basis of the molecular phylogeny of small subunit ribosomal RNA, a major taxonomic change has been proposed [8]. Arbuscular mycorrhizal fungi were removed from Zygomycota and placed into a separate monophyletic phylum, the Glomeromycota. This triggered a further subdivision of Zygomycota as part of a comprehensive phylogenetic classification of the kingdom Fungi [9]. Acknowledging the high diversity of the phylum, taxa conventionally attributed to Zygomycota were distributed among separate high-level taxa. As no overarching phylum could be determined, the groups were classified as four subphyla, i.e., Entomophthoromycotina, Mucoromycotina, Kickxellomycotina and Zoopagomycotina [10]. Using traditional taxonomic characteristics in addition to phylogeny, Humber [11] raised the Entomophthoromycotina to the phylum level as Entomophthoromycota, containing three classes (Basidiobolomycetes, Neozygitomycetes and Entomophthoromycetes). Gryganskyi et al. [12] confirmed that Entomophthoromycota is a monophyletic group and is not closely related to any of the flagellate aquatic fungi (i.e., Chytridiomycota or Blastocladiomycota). The study was later elaborated with five genes (LSU, SSU, RPB2, mtSSU, ITS), enabling positioning of the entomophthoralean fungi as a monophyletic group basal to the Mucorales [13]. Five generic lineages were recognised, *Batkoa*, *Basidiobolus*, *Conidiobolus*, *Entomophthora* and *Zoophthora*. Spatafora et al. [14] analysed genome data with 192 proteins for 46 taxa including 25 zygomycetes and proposed two further phyla, Mucormycota and Zoopagomycota. This upgrading of the classical groups of Zygomycota during the last two decades underlines the high phenotypic and phylogenetic diversity of these fungi, despite the relatively low number of extant species when compared with, e.g., the Ascomycota.

*Basidiobolus* and *Conidiobolus* are sister genera but at large phylogenetic distance, both containing members that are able to cause human infections [15]. *Basidiobolus* was introduced in the late 19th century [16] and contains ubiquitous species residing in (sub)tropical marshes. They are commonly isolated from intestines and the dung of amphibians and reptiles [17], and also occur in the intestines of warm-blooded vertebrates, including humans [15,18]. Based on LSU, SSU, RPB2, mtSSU and ITS sequences, six species are currently distinguished: *B. haptosporus, B. heterosporus*, *B. magnus*, *B. meristosporus*, *B. microsporus* and *B. ranarum* [17]. However, studies of antigens and restriction enzyme profiles suggested that all human-pathogenic isolates belong to a single species, *B. ranarum* [19,20]. The taxonomy of *Basidiobolus* is a much-debated issue. Unlike many other filamentous fungi, only few phenotypic characteristics are available to differentiate *Basidiobolus* species. The genus was previously classified in the order Entomophthorales, but was recently assigned to the order Basidiobolales because of its unresolved position close to the flagellated genus *Olpidium* [9,21].

The recent report of gastrointestinal basidiobolomycosis from Saudi Arabia [7] was carefully analysed using 28S rDNA gene sequences of five strains. These were found to cluster in a monophyletic branch close to *B. haptosporus*, having 99.97% similarity with the latter species and slightly lower (99.25%) with *B. ranarum.* Given the low variability of the used marker, these results suggest that possibly an undescribed *Basidiobolus* species was concerned. Here, we report the isolation of a similar *Basidiobolus* species from a human infection in Oman obtained with two strains from different anatomical sites of a single GIB patient. We introduce it as a novel *Basidiobolus* species, characterised by morphological features and a deviating multilocus sequence profile.

## 2. Materials and Methods

### 2.1. Phenotypic Studies

Fungal strains were isolated from a thrombus that was extracted from the common femoral artery, and from urine. Both strains were sent to the Centraalbureau voor Schimmelcultures (CBS) reference collection housed at the Westerdijk Fungal Biodiversity Institute, Utrecht, The Netherlands and deposited under the accession numbers CBS 146281 and CBS 146282, respectively. The strains were cultured on malt extract agar (MEA; Oxoid, Basingstoke, UK), potato dextrose agar (PDA; Oxoid) and Sabouraud glucose agar (SGA; Merck, Darmstadt, Germany). Culture plates were incubated at 25 °C and 30 °C for 2–5 days. Slides were examined with a Nikon Eclipse 80i light microscope, and pictures were taken using a camera attached to the microscope (Nikon; digital-sight DS-5 M, (Nikon Corporation, Tokyo, Japan).

### 2.2. DNA Amplification and Sequencing

For molecular identification, DNA was extracted from a fresh culture grown on MEA plates using the cetyltrimethyl ammonium bromide (CTAB) protocol of Möller et al. [22]. The rDNA internal transcribed spacer region (ITS) and the large ribosomal subunit (LSU) were amplified using the primer pairs ITS1/ITS4 and LR0R/LR5 [23,24,25]. Sequencing was carried out in both strand directions using the same primer pairs on an Applied Biosystems, Hitachi 3730xl DNA analyser (Applied Biosystems, Foster City, CA, USA). Consensus sequences were assembled using Seqman Pro v. 10.0.1 (DNASTAR, Madison, WI, USA). All newly generated sequences were submitted to GenBank (Table 1).

### 2.3. Phylogenetic Inference

Sequence alignments for ITS and LSU regions were generated using MAFFT v7 [26] and analysed using Maximum Likelihood (ML) and Bayesian (BI) algorithms in the CIPRES Science Gateway portal (www.phylo.org, accessed on 18 December 2020) [27]. Maximum Likelihood analyses were run using RAxML v8.2.10 (randomised accelerated maximum likelihood for high performance computing) [28] with default parameters, and node support values were estimated using rapid bootstrapping (BS) with the number of iterations set automatically by the software. Bayesian analyses were run using MrBayes v3.2.6, [29,30] and consisted of four parallel runs of 5 million generations starting from a random tree topology, sampling trees every 1000 generations. Evolutionary models for each dataset were estimated using MrModeltest v2.3 [31]. The 50% majority rule consensus trees and posterior probability (PP) values were calculated after discarding the initial 25% of saved trees as the ‘burn-in’ fraction. Individual gene phylogenies were compared to search for conflicts between significantly supported clades (ML-BS ≥ 70%, BI-PP ≥ 0.95), after which the two datasets were concatenated and analysed [32,33].

### 2.4. Antifungal Susceptibility

Antifungal susceptibility testing (AFST) of CBS 146281 and CBS 146282 was performed using broth microdilution as described in the CLSI document M38-A2. [34]. The following drugs were used; amphotericin B (Sigma-Aldrich, St. Louis, MO, USA), fluconazole (Pfizer, Groton, CT, USA), itraconazole (Janssen Pharmaceutica, Tilburg, The Netherlands), voriconazole (Pfizer), micafungin (Astellas, Ibaraki, Japan) and anidulafungin (Pfizer). Final concentrations of antifungal agents in the wells ranged from 0.016 to 16 µg/mL for amphotericin B, voriconazole, itraconazole, from 0.016 to 64 µg/mL for fluconazole and 0.008 to 8 µg/mL for anidulafungin and micafungin. Stock solutions of drugs were prepared in dimethyl sulfoxide, except for caspofungin and micafungin, which were dissolved in sterile water and stored at −80 °C until use. The strains were grown on potato dextrose agar (PDA, Difco) and incubated at 35 °C for 5 to 7 days for adequate sporulation. Three reference strains (*Paecilomyces variotii* ATCC 22319, *Candida krusei* ATCC 6258, and *Candida parapsilosis* ATCC 22019) were included as quality controls.

## 3. Case Report and Results

### 3.1. Case Presentation

A 20-year-old Omani male with type I diabetes mellitus presented to a local hospital with a two-week history of progressively worsening abdominal pain. A computed tomography (CT) scan of the abdomen revealed a complex cecal mass adherent to the pelvic floor and to the terminal ileum with multiple hepatic lesions. The patient underwent an exploratory laparotomy which revealed cecal perforation, an inflammatory mass at the base of the mesentery of the transverse colon, and extensive cecal, terminal ileum and infrahepatic inflammatory processes. Diverting loop ileostomy and drainage of the pelvic collections were performed and biopsies from multiple sites were taken for histopathological examination and for cultures. Initial histopathological examination of the intraoperative samples demonstrated fungal elements with necrosis and angioinvasion, consistent with mucormycosis. The patient was treated with liposomal amphotericin 7.5 mg/kg/day in combination with caspofungin 50 mg/day. Two days postoperatively, the patient suffered acute ischemia of the right lower limb resulting from an extensive thrombus with total occlusion of the right external iliac artery. He was transferred to Sultan Qaboos University Hospital (SQUH) for further care.

Upon presentation at SQUH, physical examination showed a conscious, ill-looking patient with pallor and signs of dehydration. He was afebrile but tachycardic (pulse rate: 104/min) and tachypnoeic (respiratory rate: 22/min) with oxygen saturation of 95% in ambient air. Blood pressure was 117/66 mm Hg. Examination of the abdomen revealed a mildly distended abdomen with midline laparotomy scar, and a functioning ileostomy with a healthy stoma. On palpation, there was right-sided abdominal tenderness with no peritoneal irritation signs. Examination of the lower limbs demonstrated signs of acute ischemia of the right leg with absent femoral, popliteal, pedal and posterior tibial pulses. Other systemic examination was within normal limits. Initial laboratory investigations showed a haemoglobin of 8.9 g/dL, and a total white cell count of 16,700 cells/μL with an eosinophil count of 1000 cells/μL. Biochemistry panel was normal with the exception of alkaline phosphatase of 372 unit/L. Glycosylated haemoglobin was 5.5% (within normal range). HIV serology was negative.

**Figure 1:**
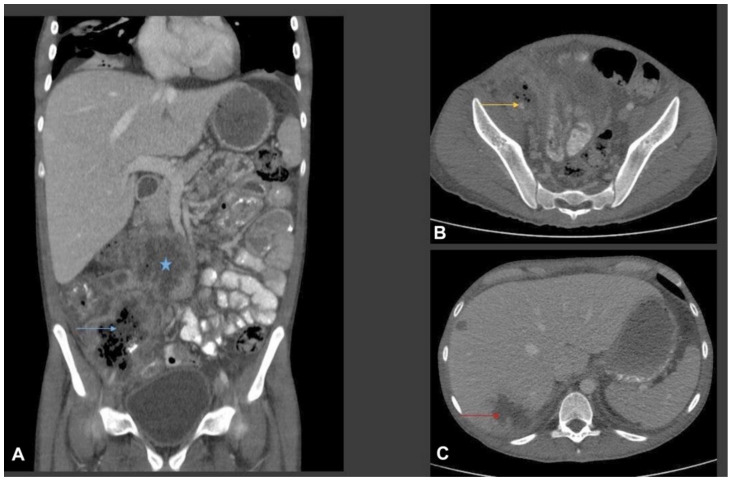
Multiplanar CT scan of abdomen with oral and intravenous contrast. (**A**) Coronal CT image shows a multispatial ill-defined low-attenuation ring enhancing collection in the root of the mesentery (star) and in the right iliac fossa. The right iliac fossa collection shows thick irregular rim enhancement with central bubbly air lucency (blue arrow). Bowel loops are adherent to the collections. There is marked inflammatory fat stranding noted in the right iliac fossa and right side of the pelvic cavity. Small volume ascites. (**B**) Axial CT image from the right iliac fossa and pelvis shows marked phlegmonous soft tissue lesion with fat stranding. The phlegmonous inflammatory mass is encasing the right external iliac artery which is small and irregular in calibre (yellow arrow). (**C**) Axial images from the liver show multifocal ill-defined low-attenuation liver lesions of variable size (two of them shown in this image). The largest was seen in the subcapsular liver in the segment VII and shows surround oedema, suggestive of liver abscess (red arrow).

The patient underwent urgent right common femoral artery thrombectomy and embolectomy. Multiplanar CT scan of abdomen and pelvis were performed with oral and intravenous contrast (Figure 1).

Then, a limited exploratory laparotomy was performed and entailed right hemicolectomy, drainage of infrahepatic collections and an abdominal wash. Samples from both surgical procedures were sent for histopathology examination and culture. High-dose liposomal amphotericin (7.5 mg/kg/day) in combination with caspofungin 50 mg/day were continued. When empirically treating cases of life-threatening mucormycosis, we use this antifungal combination based on some evidence suggesting synergistic action.

**Figure 2:**
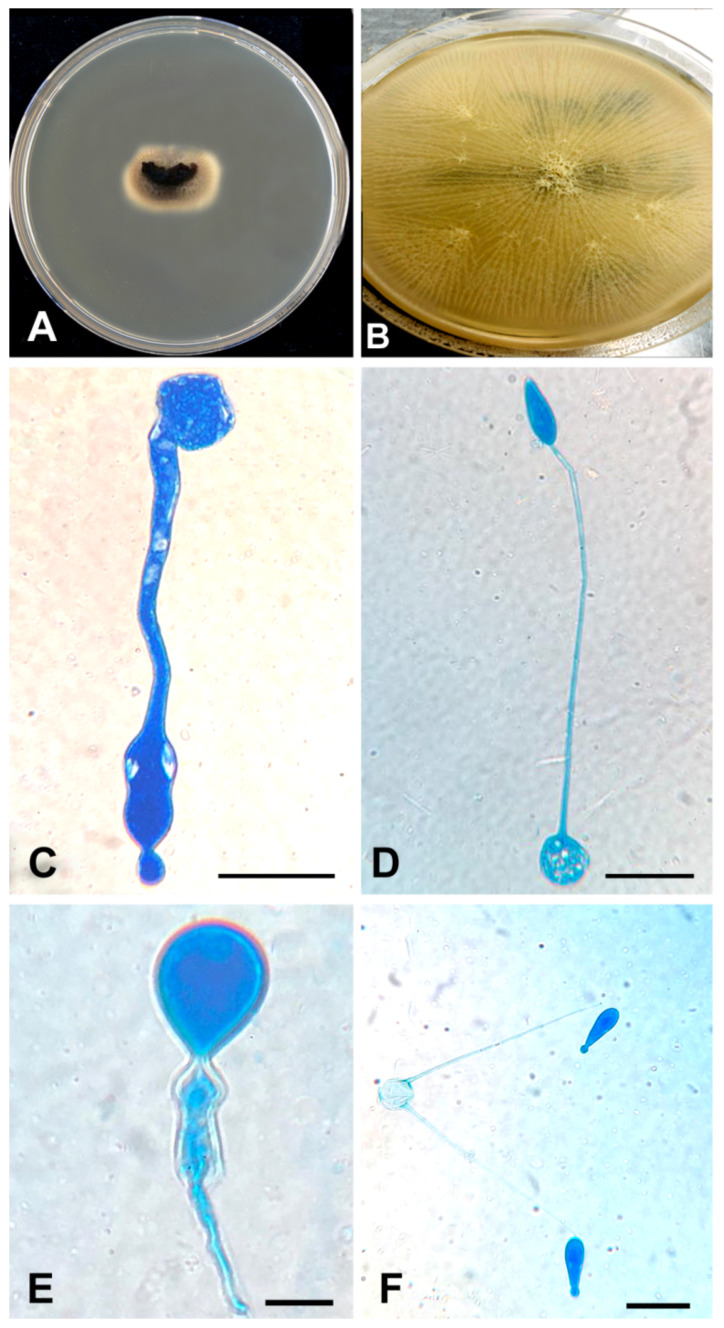
(**A**) Direct culture (SAB) from the thrombus. A piece of the thrombus (the black material at the centre) was put on a SAB plate. After incubation, the mould grew from it. (**B**) Colonial appearance of flat, radially folded, waxy, yellow-cream colonies on SAB. (**C**–**E**) Wide hyphae and club-shaped spores with knob-like tips demonstrated with lactophenol cotton blue stain. (**F**) The thin ballistoconidia in the primary culture. All scale 10 µm.

Culture of the thrombus that was extracted from the common femoral artery grew a mould that was identified phenotypically as *Basidiobolus* species (Figure 2).

**Figure 3:**
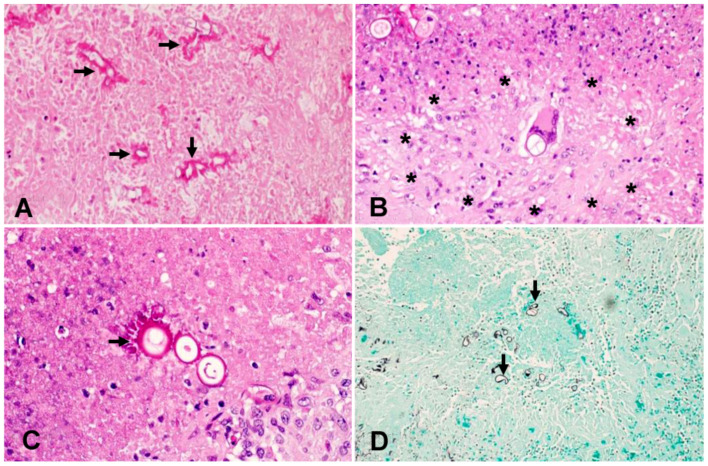
Histopathology findings of a section of small intestine: (**A**) HE stain (4×), within the necrotic areas, there are numerous fungal elements (arrows) invading all the layers beyond the mucosa. The fungi are characterised by thin walls and broad septate hyphae in keeping with basidiobolomycosis. (**B**) HE stain (40×), the fungal hyphae are associated with dense fibro-inflammatory reaction with numerous necrotising granulomas. In this image, a granuloma’s border is marked by asterisks. (**C**) HE stain (40×), hyphae are frequently surrounded by eosinophilic material known as Splendore–Hoeppli phenomenon (arrow). (**D**) GMS stain (20×), the fungi appear black with GMS stain (arrows). HE: haematoxylin and eosin; GMS: Gomori methenamine silver.

At this stage, the patient’s antifungal therapy was modified. Liposomal amphotericin was continued, but caspofungin was replaced by voriconazole (loading dose of 6 mg/kg BID for 1 day, then 4 mg/kg BID). In the absence of consensus guidelines or recommendations on the management of basidiobolomycosis, and the lack of antifungal susceptibility results for this particular isolate at this stage, this antifungal combination was chosen based on several case reports describing successful use of azoles and/or amphotericin in treating such infections. The fungal isolate was then sent to the Centre of Expertise in Mycology of Radboud University Medical Centre/Canisius Wilhelmina Hospital, Nijmegen, The Netherlands for susceptibility testing. The isolate was identified by ITS and LSU sequencing as being a novel species within the genus *Basidiobolus*. Antifungal susceptibility testing was performed according to CLSI protocols and yielded the following MIC values: amphotericin 1 µg/mL; voriconazole and itraconazole >16 µg/mL; fluconazole >64 µg/mL; anidulafungin and micafungin >16 µg/mL. As the disease progressed, the same mould also grew from multiple urine samples. Histopathology examination of the thrombus and of a section of the small intestine showed fungal elements with features compatible with zygomycetes (Figure 3).

**Figure 4:**
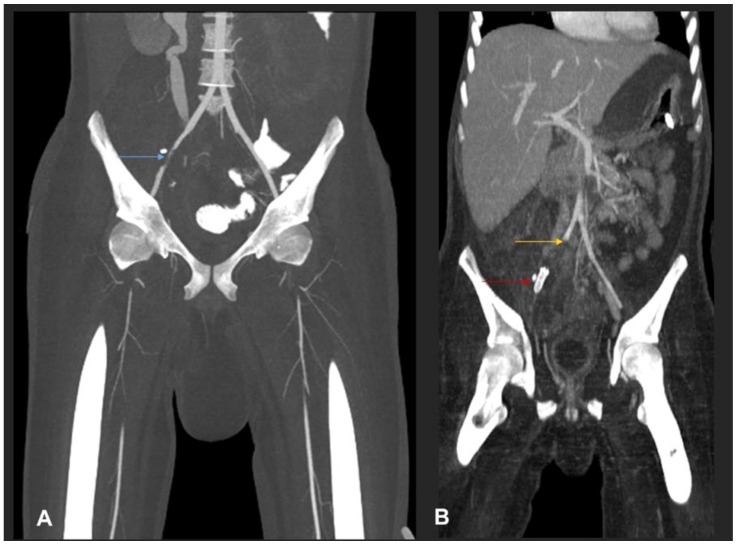
(**A**) MIP coronal CT angiography of aorta and lower limb shows a segmental narrowing and luminal irregularities of right external iliac artery (blue arrow). No occlusion. Distal run off was satisfactory. Rest of the lower limb vessels were patent and normal (not shown). (**B**) MIP coronal image of the aorta and lower limbs shows cut-off of the proximal right common iliac artery suggestive of thrombotic occlusion (yellow arrow). A stent is seen in the right external iliac artery (red arrow). Right iliac fossa shows extensive inflammatory phlegmonous mass-like soft tissue thickening.

**Figure 5:**
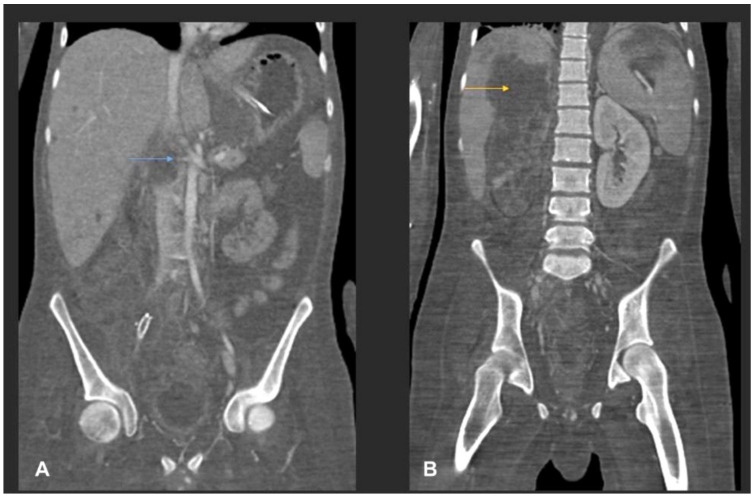
(**A**) MIP coronal images of abdomen shows a cut-off of the right proximal renal artery 1 cm from the ostium with adjacent inflammatory collection (blue arrow). (**B**) Coronal post-contrast CT shows infarction of the right kidney. A large liver abscess (yellow arrow) in the right liver lobe with extracapsular extension into the upper pole of the right kidney is also noted.

Despite the aforementioned surgical interventions and optimisation of antifungal therapy, including the empiric addition of isavuconazole (with continuation of high-dose liposomal amphotericin), the disease continued to show fulminant progression, resulting in extensive angioinvasion with involvement of the right common iliac artery, the left infrapopliteal artery and the right renal artery (Figure 4), finally resulting in bilateral lower limb ischemia and infarction of the right kidney (Figure 5).

Furthermore, the hospital course was complicated by severe sepsis secondary to extensively drug-resistant *Klebsiella pneumoniae* bacteraemia, ventilator-associated pneumonia and deep surgical site infection, culminating in his death.

### 3.2. Phylogenetic Analysis

**Figure 6:**
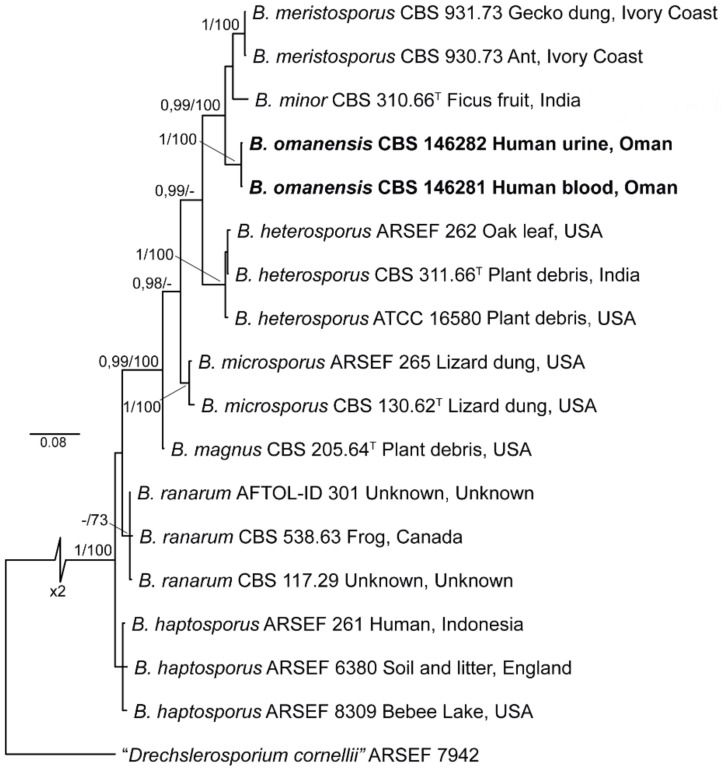
Maximum Likelihood (RAxML) phylogram obtained from the combined analysis of ITS and LSU sequences of *Basidiobolus* spp. Numbers on the nodes are Bayesian posterior probability values (BI-PP) ≥ 0.95, followed by ML bootstrap values (BS) ≥ 70%. Novel taxa proposed in this study are indicated in **bold**. Ex-type strains are indicated with ^T^. The tree was rooted to *Conidiobolus* sp. (ARSEF 7942).

The analysed sequences of CBS 146281 and CBS 146282 were found to be identical. BLAST searches of ITS sequences in GenBank matched with those of *Basidiobolus ranarum* (MK501321.1 of strain PHF-MC207) with 95.96% similarity, while LSU sequences matched with *Basidiobolus* sp. (MH256651.1, strain F43-5) with 100% similarity. For further understanding of relations between species, phylogenetic analyses were conducted using ITS and LSU sequences, which included available type and reference sequences of *Basidiobolus* spp. known from culture (Table 1). Each dataset was analysed and compared separately prior to multilocus analysis. The final concatenated alignment comprised sequences from 17 strains, including the outgroup (*Conidiobolus* sp. ARSEF 7942), and consisted of 1551 nucleotide positions (ITS = 566, LSU = 985), of which 1140 were conserved (ITS = 369, LSU = 771), 402 were variable (ITS = 197, LSU = 205) and 206 were phylogenetically informative (ITS = 97, LSU = 109) (Figure 6). Phylogenetic analysis of individual and combined loci resolved eight well-supported clades, six of which corresponded to known *Basidiobolus* species (i.e., *B. haptosporus*, *B. heterosporus*, *B. magnus*, *B. meristosporus*, *B. microsporus* and *B. ranarum*). Strains CBS 146281 and CBS 146282 clustered in a monophyletic clade, supported by high BS and PP values (1 and 99%, respectively), and are here described as a novel species, *Basidiobolus omanensis*. Additionally, the ex-type strain of *B. haptosporus* var. *minor* (CBS 310.66) clustered consistently in a well-supported linage, unrelated to the *B. haptosporus* core clade, and it is therefore elevated to species level below.

### 3.3. Antifungal Susceptibility

Antifungal susceptibility testing of *B. omanensis* was performed using broth microdilution protocols according to CLSI M38A2, resulting in the following MICs: amphotericin B 1 µg/mL; fluconazole >64 µg/mL, itraconazole and voriconazole >16 µg/mL; and anidulafungin and micafungin >16 µg/mL.

### 3.4. Taxonomy and Description

*Basidiobolus omanensis* Al-Hatmi, Sand.-Den., Balkhair, Al Adawi & de Hoog, sp. nov.—Figure 6. MycoBank MB 839872.

Etymology: ‘omanensis’ refers to the country in which the fungus was first isolated.

Holotype: Oman, Muscat, from thrombus in common femoral artery of human patient, CBS 146281, designated here, preserved in metabolically inactive condition in liquid nitrogen. Living strain derived from type CBS 146281.

**Figure 7:**
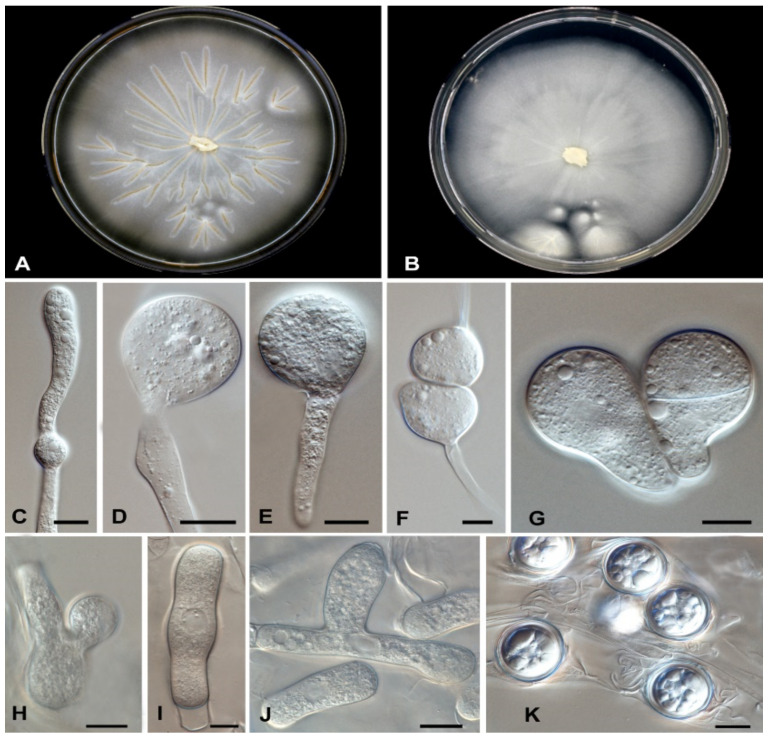
*Basidiobolus omanensis*. A colony on (**A**) MEA, (**B**) SAB after 3–5 days at 25 °C, (**C**) mycelia with young chlamydospore, (**D**) primary conidiophore arising from mycelia, (**E**) primary conidia, (**F**) formation of zygospores, (**G**) mature septate conidia, (**H**) mature zygospores, (**I**,**J**) branched and unbranched hyphae, (**K**) mature zygospores. Bars 10 μm.

Description: based on colonies of CBS 146281 grown for 5 d at 25 °C on MEA and SGA. On MEA, colonies grow rapidly, with an average daily growth rate of 5–6 mm; whitish, flat, waxy, glabrous and radially folded; margin entire, straight (Figure 7A,B). On SGA, colonies flat, membranous, with a smooth, glabrous and waxy appearance, becoming powdery in a later stage, with colourless aerial mycelia (Figure 7B). Radiate folds developed from the centre on both media (Figure 7A,B).

Hyphal elements hyaline, unbranched, coenocytic or with occasional septa, broad, 5–10 μm in diameter (2 to 4) (Figure 7I,J). Primary conidiophores arising from hyphal segments, unbranched, producing a single globose conidium, 4–9 μm diameter (Figure 7D,E). Secondary conidia discharged, colourless, globose to subglobose, 15–20 µm in diameter, with papilla and with or without septum, 3–5 × 1–4 μm (Figure 7G,H). Immature zygospores developed after the encounter of two hyphal segments, producing a swelling in the contact area and later becoming globose (Figure 7F). Smooth, thick-walled, spherical chlamydospores are present (Figure 7F). Numerous smooth, globose to subglobose, multinucleate zygospores, 2–4 um in diameter present. Some of the mature zygospores contained a globule at the centre, with some space between the internal globule and the cell wall (Figure 7K).

*Basidiobolus minor* (Sriniv. & Thirum.) Al-Hatmi, Sand.-Den. & de Hoog, comb. et stat. nov.—MycoBank MB 839873.

*Basionym*: *Basidiobolus haptosporus* var. *minor* Sriniv. & Thirum., Mycopath. Mycol. Appl. 33: 60. 1967.

## 4. Discussion

We report a newly described *Basidiobolus* species causing refractory angioinvasive gastrointestinal disease (GIB) in a 20-year-old Omani patient with type 1 diabetes mellitus. His disease seemed to have started in the caecum with extension to the major vessels of the lower limbs. Physical examination of the lower limbs demonstrated signs of acute ischemia, confirmed by imaging studies including CT (Figure 1, Figure 4 and Figure 5) and by histopathology (Figure 3). From a clinical perspective, the present case is similar in main traits to other reported cases of GIB. The infection is a rare but possibly emerging disease entity, affecting immunocompetent individuals including children, and mainly occurs in hot climates worldwide [35]. In many earlier reported cases, surgery was performed without preceding diagnosis of GIB [36].

The first case of basidiobolomycosis was a subcutaneous infection reported by Joe et al. [1] from Indonesia in 1956. Gastrointestinal basidiobolomycosis has been reported worldwide, mainly in tropical and subtropical climate zones of Asia and North America [37]. Mohammadi et al. [38] reviewed more than 102 cases of GIB published between 1997 and 2018. Many cases had been reported from Saudi Arabia (62), Iran (24) and the U.S.A. (21), and to a lesser extent from Iraq (6), Kuwait and France (two cases each), Thailand, Qatar, Oman, The Netherlands and Brazil (one case each). The disease seems to be relatively common in the Middle East. *Basidiobolus* species cause infections in both adult and paediatric populations, while most paediatric cases were reported from Saudi Arabia [39]. Subcutaneous basidiobolomycosis has highly diverse and non-specific symptoms. On the other hand, patients with GIB may present with fever, abdominal pain, chills, weight loss, diarrhoea or constipation, and an abdominal mass can be observed [38]. As *Basidiobolus* is an environmental saprobe residing in soil and decaying vegetables and fruits, the route of transmission in subcutaneous disease appears to be minor trauma resulting, e.g., from an insect bite, while GIB can be acquired via intravenous catheters, intramuscular injection or ingestion of soil or faeces via contaminated food [20]. The latter transmission route may be very common, as *Basidiobolus* has been detected as a part of the normal mycobiome inhabiting the human gastrointestinal tract [40].

Laboratory results of our patient show an elevated white blood cells count (WBCs) of 16,700 cells/µL, with an elevated eosinophil count of 1000 cells/µL, normal glycosylated haemoglobin and deranged liver function tests. These results agreed with previously reported cases where elevated WBCs and eosinophilia were observed [37]. Abdominal imaging studies such as computed tomography (CT) are often used during the evaluation of patients with GIB [37]. In a review of abdominal imaging findings in GIB, Flicek et al. [41] commonly found abdominal masses in colon, liver or multiple sites, and bowel wall thickening. Although such findings are not diagnostic of GIB, the authors concluded that in the right clinical and epidemiological context, one should suspect GIB when an abdominal mass is seen upon CT scan [41]. In our patient, abdominal CT scans revealed a complex caecal mass adherent to the pelvic floor and to the terminal ileum with multiple hepatic lesions (Figure 1). Histological criteria such as a granulomatous reaction and mycological evidence of fungal structures are taken as suggestive of GIB [42,43]. Detailed examination of the thrombus and a section of the small intestine of our patient showed thin-walled, broad and septate hyphae, and these features are compatible with zygomycete-like fungi (Figure 3). Diagnosis of *Basidiobolus* infection down to the species level is typically accomplished by microbiological culture as gold standard [44]. From the thrombus specimen that was extracted from the common femoral artery, and from a urine sample, a mould was grown reminiscent of a *Basidiobolus* species. The colonies on PDA were expanding, subhyaline, waxy and without aerial mycelium. The wet mount preparation showed morphological characteristics consistent with *Basidiobolus* by production of zygospores with smooth walls, and retained short, paired protuberances leading to a structure known as “beaked zygospore”. In addition, apical, globose primary conidia were produced with forcible conidium discharge from the conidiophores, and with pyriform secondary conidia (Figure 2 and Figure 7). The thin ballistoconidia were seen only in the primary culture (Figure 2F). Overall, distinct morphological traits are minimal or absent between *Basidiobolus* species. Most of the *Basidiobolus* species had been distinguished on the basis of phenotypic differences such as the form of zygospores, formation of aerial hyphae, production of exogenous microspores and odour production during growth, as well as growth temperature preferences [18].

BLAST searches of the rDNA sequences of CBS 146281 and CBS 146282 revealed that the ITS sequences matched a strain of *Basidiobolus ranarum* with 95.96% similarity, while LSU sequences matched 100% with an unnamed *Basidiobolus* species. The large ITS distance to a described species, and judging from the phylogenetic ribosomal tree, led to the conclusion that our isolate is taxonomically separate from the recognised species of *Basidiobolus* (Figure 6) and that no formal name is available for this taxon. The new species *B. omanensis* is morphologically similar to other *Basidiobolus* species occurring in humans. The genus currently includes seven species, namely *B. haptosporus*, *B. heterosporus*, *B. magnus*, *B. meristosporus*, *B. microsporus*, *B. minor* and *B. ranarum*. In this study, *B. omanensis* is added as a novel species. Previously, only *B. ranarum* was reported to cause human infection, due to the fact that in most published cases, *B. haptosporus* and *B. heterosporus* have been regarded as synonyms of *B. ranarum* [45]. A previous study showed that a set of *Basidiobolus* spp. from Saudi Arabia clustered as a novel monophyletic lineage [7]. According to the same authors, these strains shared 99.97% ITS similarity with *B. haptosporus* and 99.97% with *B. haptosporus* var. *minor*, and lower similarity with *B. ranarum* (99.93%), a species which is commonly linked to GIB [7]. The latter results suggest the discovery of a new and serious causal agent of GIB. Thus, our results strongly support the recognition of *B. omanensis* isolates as a novel species within the genus *Basidiobolus*; the differences observed for all phylogenetic markers are considered sufficient to propose *B. omanensis* as a new species of *Basidiobolus*. The phenotypic characteristics proposed in the literature provide insufficient resolution to differentiate the seven known species of *Basidiobolus*; these characters mostly include odour production along with physiological features [46,47,48,49]. However, these variations have not been systematically evaluated in larger numbers of strains. Sequence-based diagnostics are therefore required. Morphologically, the new species *B. omanensis* is similar to remaining species in terms of colonies with satellites, conidia, conidiophores, zygospores and chlamydospores.

## Figures and Tables

**Table 1:** Strain information and accession numbers of DNA sequences included in the phylogenetic analyses.

Species Epithet	Strain ^1^	Host/Substrate	Country	Sequence Accession Number ^2^	
				ITS	LSU
***B. haptosporus***	**ARSEF 261 = NRRL 1232 = RSA 228**	**Human**	Indonesia	EF392520	MH869969
	ARSEF 6380	Soil and litter	UK	EF392528	MH869969
	ARSEF 8309	Unknown	USA	EF392531	EF392420
*B. heterosporus*	ARSEF 262 = RSA 964	Oak leaf	USA	EF392521	EF392411
	CBS 311.66 = ATCC 16580 = IMI 118288 = NRRL 3687 (ex-type)	Plant debris	India	MH858801	JX242587
*B. magnus*	CBS 205.64 = ATCC 15379 = NRRL 3734 (ex-type)	Plant debris	USA	NR_077175	EF392423
*B. meristosporus*	CBS 931.73	Gecko dung	Ivory Coast	**MT830914**	**MT831974**
	CBS 930.73	Ant cemetery of *Paltothyreus tarsatus*	Ivory Coast	**MT830915**	**MT831975**
*B. microsporus*	ARSEF 265 = RSA 962	Lizard dung	USA	EF392523	DQ364202
	CBS 130.62 = ATCC 14708 = DSM 3120 = IMI 093345 = RSA 977 (ex-type)	Lizard dung	USA	NR_159603	MH869698
*B. minor*	CBS 310.66 = ATCC 16579 = IMI 118287 = NRRL 3677 = NBRC 109014 (ex-type)	*Ficus* fruit	India	EF392535	EF392424
*B. omanensis*	CBS 146281 (ex-type)	human intravascular thrombus	Oman	**MT830913**	**MT831973**
	CBS 146282	Human urine	Oman	**MT830912**	**MT831972**
*B. ranarum*	AFTOL-ID 301	Unknown	Unknown	AY997030	DQ273807
	CBS 538.63	Frog	Canada	MH858348	MH869969
	CBS 117.29 = ATCC 11230 = IFO 9117	Unknown	Unknown	IF00911701 ^a^	IF00911701 ^a^
*Conidiobolus* sp.	ARSEF 7942	Leaf litter	USA	EF392537	KC788411

^1^ AFTOL-ID = Assembling the Fungal Tree of Life, Department of Biology, Duke University, NC, USA; ARSEF = ARS Collection of Entomopathogenic Fungi, USDA-ARS BioIPM Research Unit, Ithaca, NY, USA; ATCC = American Type Culture Collection, Manassas, VA, USA; CBS = Westerdijk Fungal Biodiversity Institute, Utrecht, The Netherlands; DSM = German Collection of Microorganisms and Cell Cultures GmbH, Braunschweig, Germany; IFO = Institute for Fermentation, Osaka, Japan; IMI = CABI Bioscience, Egham, Surrey, UK; NBRC = NITE Biological Resource Center, Chiba, Japan; NRRL = ARS Culture Collection, USDA-ARS National Center for Agricultural Utilization Research, Peoria, IL, USA; RSA = Rancho Santa Ana Botanic Garden, Claremont, CA, USA. ^2^ Unless otherwise specified, accession numbers refer to GenBank. ^a^ Accession numbers from NBRC sequence database. Newly generated sequences are highlighted in **bold**.

## Data Availability

All sequences generated in this study were submitted to GenBank.
